# A qualitative inquiry on pregnant women’s preferences for mental health screening

**DOI:** 10.1186/s12884-017-1512-4

**Published:** 2017-10-03

**Authors:** Hamideh Bayrampour, Deborah A. McNeil, Karen Benzies, Charleen Salmon, Karen Gelb, Suzanne Tough

**Affiliations:** 1 Department of Family Practice, Midwifery Program, University of British Columbia, 3rd Floor David Strangway Building, 320–5950 University Boulevard, Vancouver, BC V6T 1Z3 Canada; 2 Alberta Health Services and Associate Professor in Faculty of Nursing and Department of Community Health Sciences Cumming School of Medicine, University of Calgary, Calgary, T2W 3N2 Canada; 3 Professor in Faculty of Nursing, University of Calgary, Calgary, T2N 1N4 Canada; 4 Department of Community Health Sciences, Cumming School of Medicine, University of Calgary, Calgary, T2N 1N4 Canada; 5 Midwifery Program, Department of Family Practice, Faculty of Medicine, University Boulevard, Vancouver, BC V6T 1Z3 Canada; 6 Alberta Innovates Health Solutions Health Scholar and Professor in Department of Pediatrics and Community Health Sciences, University of Calgary, Calgary, T3B 6A8 Canada

**Keywords:** Pregnancy, Mental health screening, Depression, Anxiety, Prenatal care

## Abstract

**Background:**

Approaches to screening can influence the acceptance of and comfort with mental health screening. Qualitative evidence on pregnant women’s comfort with different screening approaches and disclosure of mental health concerns is scant. The purpose of this study was to understand women’s perspectives of different mental health screening approaches and the perceived barriers to the communication and disclosure of their mental health concerns during pregnancy.

**Methods:**

A qualitative descriptive study was undertaken. Fifteen women, with a singleton pregnancy, were recruited from a community maternity clinic and a mental health clinic in Calgary, Canada. Semi-structured interviews were conducted during both the 2nd and 3rd trimesters. Data were analyzed using thematic analysis.

**Results:**

Preferences for mental health screening approaches varied. Most women with a known mental health issue preferred a communicative approach, while women without a known mental health history who struggled with emotional problems were inclined towards less interactive approaches and reported a reluctance to share their concerns. Barriers to communicating mental health concerns included a lack of emotional literacy (i.e., not recognizing the symptoms, not understanding the emotions), fear of disclosure outcomes (i.e., fear of being judged, fear of the consequences), feeling uncomfortable to be seen vulnerable, perception about the role of prenatal care provider (internal barriers); the lack of continuity of care, depersonalized care, lack of feedback, and unfamiliarity with/uncertainty about the availability of support (structural barriers).

**Conclusions:**

The overlaps between some themes identified for the reasons behind a preferred screening approach and barriers reported by women to communicate mental health concerns suggest that having options may help women overcome some of the current disclosure barriers and enable them to engage in the process. Furthermore, the continuity of care, clarity around the outcomes of disclosing mental health concerns, and availability of immediate support can help women move from providing “the best answer” to providing an authentic answer.

## Background

Depression and anxiety are common during pregnancy. Prevalence estimates of 6% and 17% have been reported for major and minor depression [1], respectively. The rates of anxiety symptoms and anxiety disorders during the antenatal period are 23% [2] and 15% [3], respectively. Poor maternal mental health is linked to several adverse outcomes including preterm birth [4, 5], postpartum depression [6, 7], difficulty in attachment and bonding, cognitive and developmental delays and mental health problems in children [8–12]. Perinatal depression and anxiety carry significant long-term costs to society related to adverse impacts on child behavioral and developmental outcomes and women’s quality of life [13].

Women with perinatal mental health problems often do not seek help [14]. The benefits of perinatal mental health screening include early detection and management that can reduce symptoms and their severity [15]. Professional organizations recognize the risk of poor mental health during perinatal period and the potential benefits of screening [16, 17]. The National Institute for Health and Care Excellence (NICE) clinical guideline on antenatal and postnatal mental health recommends screening for both anxiety and depression during pregnancy [16]. The American College of Obstetricians and Gynecologists recommends that clinicians screen women at least once during the perinatal period for depression and anxiety symptoms [18].

Screening acceptability is individuals’ willingness to complete a questionnaire or conduct a procedure [19]. Quantitative data show that most women are comfortable with perinatal mental health screening. However, some women are not able to confide in their prenatal care providers about mental health issues and are unwilling to disclose and share how they feel [20, 21]. One in five pregnant women reports being somewhat honest or not at all honest during mental health screenings [22]. Additionally, the women who could benefit most from screening frequently are those who are hesitant or skeptical towards screening. In a Canadian survey, 17% of pregnant women identified at least one harm pertaining to screening, such as feeling embarrassed, feeling worried about what would happen with the information, not knowing why certain questions were asked, finding the experience to be negative, and finding the questions or the way questions were asked uncomfortable. A previous diagnosis of a mental health issue was more common among women who identified harms associated with screening than among those who did not (38% vs. 22%) [23].

The screening method is part of screening acceptability [19]. Quantitative evidence suggests that approaches to perinatal mental health screening may influence women’s comfort with screening [19]. However, associations between comfort with screening and the ability to be honest have been reported only for certain screening approaches such as telephone-based screening [22]. Qualitative evidence on women’s comfort with different screening approaches and disclosure of mental health concerns during pregnancy is scant. Because the ultimate goal of screening programs is to identify those with poor mental health, an in-depth inquiry is needed to understand the reasons for preferring a certain approach and how these approaches affect women’s comfort and ability to be honest and disclose mental health concerns. Given the accumulating evidence on the significance of maternal mental health [4, 5, 8–12] and the recognition of importance of implementing screening programs within primary care settings [17], gaining an understanding about the acceptability of various screening approaches is essential. This evidence can inform design and implementation of screening programs for those most in need of assistance, particularly considering the stigma around mental health [14]. The purpose of this study was to understand women’s perspectives of different mental health screening approaches and the perceived barriers to the communication and disclosure of their mental health concerns during pregnancy.

## Methods

A qualitative descriptive study [24] was conducted. Using purposeful sampling [25], participants were recruited from a community maternity clinic, and a mental health clinic in Calgary, Canada to ensure diversity of women’s mental health condition. Inclusion criteria included women being 20 years or older, having a singleton pregnancy at the second trimester of pregnancy (20–24 weeks), and ability to read, write, and speak in English. Each woman participated in a semi-structured interview at 2nd trimester of pregnancy and was invited to a second interview at 3rd trimester. Interviews were conducted by two female interviewers (HB; a postdoctoral fellow with prior qualitative research experience and an interest in maternal mental health and CS; an undergraduate student with no prior research experience; CS conducted two interviews that were followed by a supplementary interview by HB). The interviewers did not know the participants prior to the first interview. Semi-structured interviews were conducted using an interview guide adapted from a previous study [26]. The adopted interview guide was pilot tested and the pilot interview data were not included in data analysis. The clinics’ staff identified potential participants. Face-to-face interviews were conducted at the clinics between November 2012 and July 2013. Data collection continued to the point of data saturation [27] when no new information or themes were identified. At the first interview, a brief questionnaire was used to collect socio-demographic information. Interviews were audio-recorded and transcribed verbatim. The study was approved by the Conjoint Health Research Ethics Board (E-24854) and participants signed a written informed consent form.

### Data analysis

Data analysis was carried out concurrently with data collection using thematic analysis [28]. Each interview was read in full by researchers independently and then analysis proceeded with open coding in the margins of the transcripts. A definition of each code was developed and codes were clustered into categories, and finally the categories were grouped into themes. The research team met regularly to discuss and reach consensus on coding, coding scheme, and themes. Data analysis was managed using NVivo. Descriptive statistics were used to describe the characteristics of the participants using IBM SPSS Statistics version 19.0.0.1 [29].

The essences of rigor in qualitative inquiry are the visibility of research practices and accountability of the data analysis [30, 31]. In the current study, credibility was established by member checking, independent analysis of data by three researchers (HB, DM, KB), peer review and debriefing through regular meetings with the research team to achieve a jointly developed interpretation of the data, and reporting verbatim quotes. Dependability and confirmability were obtained through the use of an audit trail, documenting contextual information, and participants’ reflections, and reporting detailed methodological descriptions. Transferability was demonstrated by providing details of study participants to clarify the characteristics of the women to enable readers to identify applicability to populations with whom they work.

## Results

Of the 18 women invited to participate, 15 agreed to take part in the study. Sample characteristics are presented in Table 1. Of 15 participants, 12 (80%) took part in the second interview conducted at the 3rd trimester. In total, 27 individual interviews, each about one hour, were conducted. The mean gestational age at the first interview was 21.8 (±2.2) weeks and at the second interview was 34.4 (±1.9) weeks. Four participants had medical complications during the current pregnancy and six experienced mental health problems in the past, of which four had received treatments (Table 2). The findings are presented in two sections: (1) screening preferences and (2) barriers for communicating mental health concerns.

**Table 1:** Demographic Characteristics of Participants (*N* = 15)

Variables	M (SD)
Age (years)	30.93 (4.56)
Gestational age at recruitment (weeks)	21.80 (2.15)
	n (%)
Nulliparous	7 (46.7)
Multiparous	8 (53.3)
Marital Status	
Married	11 (73.3)
Common-law or live-in partner	3 (20.0)
Single	1 (6.7)
Education	
High school**/**incomplete university	3 (20.0)
Diploma/certificate (e.g. hygienists)	5 (33.3)
Bachelor’s degree	7 (46.7)
Household income	
Under $20,000	2 (13.3)
$40,000 - $79,000	4 (26.7)
$80,000 and above	9 (60.0)
Racial/ethnic background	
Other	4 (26.7)
White (Caucasian)	11 (73.3)
Family Doctor	
Yes	14 (93.3)
No	1 (6.7)
Paid work before current pregnancy	
Yes	14 (93.3)
No	1 (6.7)
Paid work during current pregnancy	
Yes	10 (66.7)
No	5 (33.3)
Born in Canada	
Yes	12 (80.0)
No	3 (20.0)

**Table 2:** Health Characteristics of Participants (*N* = 15)

Variables	n (%)
Thinking back to before you were pregnant, would you say you wanted to be pregnant …	
Sooner	3 (20.0)
Later	6 (40.0)
Then	6 (40.0)
Not at all	0 (0.0)
Problems or complications during pregnancy (at time of recruitment)	
Yes	4 (26.7)
No	11 (73.3)
Experienced any previous mental health problems (e.g. depression, generalized anxiety, bipolar disorder, etc.)	
Yes	6 (40.0)
No	9 (60.0)
Treatment for mental health problems	
Yes	5 (33.3)
No	1 (6.7)
No problems with mental health indicated	9 (60.0)
History of mental health problems in family	
Yes	4 (26.7)
No	11 (73.3)

### Screening preferences

During the interviews, participants were asked about their screening preferences and reasons for favoring certain approaches. Preferences for mental health screening varied, and there was no consensus among participants on a preferred format. All except one participant indicated the same preferred method at both the 2nd and 3rd trimester interviews. The findings for screening preferences and motives were categorized into two groups: less interactive approach and communicative/interactive approach.

### Less interactive approach

Less interactive approaches included any screening conducted through a self-reported questionnaire completed in a clinic or online. The reasons for preferring this approach, in addition to convenience, included thinking things through, sustaining personal space and not revealing emotional vulnerability.

#### Thinking things through

A need to think about answers emerged from the interviews with women who preferred this approach. Most participants explained that this approach would give them time to think and to ensure they answered questions accurately, while, others reported that this approach would give them time to think about the answer that they wanted to share. A nulliparous woman commented:

“I can think about what people say to me and I can think about what I want to say back. Whereas a phone conversation is you gotta be quicker than that.” (Participant 6)

Some women indicated that they were cautious about how to respond to the questions and would need extra time to provide “the best answer”. A 32-year-old woman struggling with obsessive thoughts commented:

“The direct asking the questions I think people might need a bit of time. For me, I’m careful how I like to answer because I’d like to give the best answer kind of thing so... I need a little bit of time to kind of think of what I need to say. For me, the best way is to say while you’re here we need you to fill out this questionnaire.” (Participant 12)

#### Sustaining personal space and not revealing emotional vulnerability

Women, who preferred a less interactive format for screening, often reported a tendency to conceal their emotions for variety of reasons such as not wanting to expose emotions or be seen vulnerable. Some participants stated that they would evade the conversation altogether if they thought they might become emotional. A multiparous woman who was struggling to choose the mode of delivery after a caesarean birth and was fearful to make a decision commented, “If I don’t show anybody that I’m scared, then no one will know that I’m scared.” She explained:

“I’ve always kind of have been viewed as the stronger person [voice cracks at the words “stronger person” and starts crying] [pause]. I uh yeah really hate being emotional in front of other people, so I think that’s probably why. I think, if I feel like I’m going to get emotional, I will avoid a topic, so I uh do not want people to see that weakness cause everybody kind of just thinks like someone once called me the Margaret Thatcher once.” (Participant 5)

Hence, sustaining personal space also emerged as these women felt that in less interactive approaches, particularly online screening, they would have their own space if they become emotional. A Nulliparous woman commented:

“That’s online if I’m emotional and I don’t want other people to know then they don’t have to know and I kind of be in my own space. And [that is] the nice thing about being online.” (Participant 6)

### Communicative approach

Communicative approaches included any screening performed through communication with a health care provider, such as an in-person or telephone-based screening. In this category, all but one participant preferred an in-person format. The reasons for preferring this approach included being a personal interaction, helping to talk it through, and allowing the instant exchange of feedback.

#### Being a personal interaction

The participants who favored this approach indicated that they would prefer a personal interaction to connect with their provider in order to communicate mental health concerns. A 26-year-old multiparous participant commented, “you can’t really connect with a piece of paper.” Some women also explained that despite the discomfort of becoming emotional during a communicative approach, it would be easier to explain concerns in-person.

#### Helping to talk it through

Women, who preferred this approach, expressed a need to ensure that they were communicating their emotions clearly and “getting their point across” so their care provider was able to evaluate their condition more accurately. They noted that unlike a questionnaire with close-ended questions, an in-person conversation would provide an opportunity to explain their concerns more thoroughly and to elaborate for further clarification if necessary. A nulliparous woman commented:

“Sometimes it’s just multiple choice and none of them really feel like they fit and I don’t want to have to find an answer or to write it to explain myself either it becomes really long or I’m not sure that I’m getting my point across. So I think talking to someone verbally whether it’s on the phone or in person – there’s no difference – at least I feel confident that they get my point across and they can properly evaluate.” (Participant 6)

Some participants also indicated that this approach would help them understand their own feelings. A 22 year-old nulliparous woman commented:

“I would be an in-person kind of, yeah. Because when you start chatting you realize things that you wouldn’t when you write it down. Umm I feel like you … like when the feelings start coming out that you don’t realize when you’re talking in person. So definitely I think that it’s important to have those kind of conversations and make them personal.” (Participant 11)

#### Allowing the instant exchange of feedback

Participants favoring this approach indicated that through a communicative screening, they would derive meaning, gain clarity from sharing their concerns, and potentially obtain strategies to address their mental health challenges. Also from some participants’ quotes, it appeared that this approach would provide an opportunity for a two-way information exchange. A nulliparous woman who had a history of depression and described herself as skeptical with regard to trusting people reported that actually talking to a person and receiving non-verbal and social cues would also help her to know the provider better when discussing her mental health concerns. She commented:

“I’m kind of old fashioned when it comes to this stuff. I’m about social interaction with an actual person as opposed through a computer. I find that very impersonal so for me, its just like, the actual talking to a person and being able to see social cues and you know the non-verbals and things along that line so you get to know a person a little bit better, easier…its just nice to get that feedback.” (Participant 3)

### Barriers to communicating mental health concerns

We asked participants whether they would share their mental health concerns with their maternity care providers. Some participants reported that they would communicate these concerns and would seek support if they needed it; however, others indicated that they would not. The reasons for not sharing mental health concerns with a health care provider were classified into internal (personal) barriers and external (structural) barriers.

### Internal barriers

Internal barriers included a lack of emotional literacy, fear of disclosure outcomes, feeling uncomfortable to be seen vulnerable, and perception about the role of prenatal care provider.

#### Lack of emotional literacy

A lack of emotional literacy that is the knowledge about mental health issues and treatments [32] impeded communicating mental health concerns. Two sub-themes emerged for this theme including not recognizing the symptoms and not understanding the emotions.

##### Not recognizing the symptoms

According to the interviews, some women did not realize they had mental health problems due to unfamiliarity with symptoms of mental health issues. A participant who was expecting her third child noted that she was not aware that she was struggling with anxiety until she filled out a screening form at a vaccination clinic. This 37-years-old woman indicated that it was the first time that she could find a word to describe how she was feeling:

“It was the first time that I could find a descriptive word that could match how I was feeling. It was like ‘panicky to take him somewhere or drive him somewhere’ and just in general checking up around him during the night and things like that… I recognized that I was panicky versus concerned then I realized that I had you know my feelings were coming from a place of fear versus a place that’s probably more normal.” (Participant 7)

##### Not understanding the emotions

Most women reported that if they were not able to understand emotions themselves they would not communicate it. A 22-year-old single mother with an unplanned pregnancy explained that she was not ready to share her feelings because she wanted to keep her emotions inside until she had a better understanding of them. She commented:

“I think I was just so emotional that it was just better to keep it all inside and figure out and try to understand what was going on and try to understand … I just felt that like my emotions were all over the place, all the time. And I just didn’t understand them. I’m probably still like that.” (Participant 11)

A number of women also reported not communicating their emotional struggles because they were uncertain whether these concerns were normal and whether other women also experience these struggles.

#### Fear of disclosure outcomes

Participants’ explanations reflected that the fear of disclosure outcomes hindered communicating their emotional concerns with the provider. Two sub-themes were identified for this theme including fear of being judged and fear of the consequences.

##### Fear of being judged

Fear of being misunderstood and judged emerged as reasons for not sharing concerns. These fears were particularly evident when the women did not know whether their experiences were normal. A 32-year-old pregnant woman who was suffering from obsessive thoughts of harming her toddler explained that because the providers did not know her and her values as a person, they might misunderstand this information. She explained:

“I’d say that I’m a little bit reserved with anybody that I don’t know especially well… I would say with my husband I try to be as honest as possible because we need to know how we’re feeling and I see him every day and stuff but someone that I just see once a week or once every month kind of thing, I don’t know what ... how they’ll perceive what I’m saying to them or how they’ll take it as a misunderstanding you know? Like I’m gonna harm somebody like my baby? What if they think I’m gonna you know... they don’t know my you know... they don’t know my personal values and they don’t know me” (Participant 12)

In response to the question regarding how the prenatal care provider could address this concern, this participant noted that presenting some examples would be helpful:

“Like the doctor [says something] like ‘this is what I hear from another person’ and... ‘this is what I was thinking when this mother said this to me’.” (Participant 12)

It also appeared that this presentation of real-world examples could be helpful for women with mental health concerns in general:

“Like it’s normal to have these types of feelings and it’s not different than any other type of pregnancy. I just want peace of mind and … be able to sleep at night.” (Participant 15)

##### Fear of the consequences

Being reserved and skeptical about sharing mental health struggles due to fear of potential consequences was a reoccurring concept. A 26-year-old woman with a history of anxiety disorders described communicating emotional concerns with a health care provider as talking to “a complete stranger”. She commented:

“…Because you have no idea who this person is. So you know it’s different than talking to your mom or sister about something. Um not that I’d talk to them about this but then talking to a complete stranger whose *job* is to just sit there and decide something about you.” (Participant 14)

A need to know what the care provider would do with disclosed information and whether it would involve further communication to clarify the normality of feelings or more serious social services interventions also emerged. The participant with obsessive thoughts expressed that she would not share her concerns due to the uncertainty about the outcomes of this disclosure:

“There is that kind of thing where you’ll be conservative kind of thing to discuss with your doctor... because if you ask the question and leave it at that like you don’t know if they’re gonna call social services on me …Like, ‘Well, these are normal feelings to have right now’ or… ‘How do you feel about that right now because I have a car waiting outside to take your baby away’.” (Participant 12)

#### Feeling uncomfortable to be seen vulnerable

Some participants reported being uncomfortable to be seen vulnerable. They perceived revealing emotional problems and asking for help as a sign of weakness. Some women explained that it was difficult for them to expose their emotions and become emotional. A 32-year-old participant in her second pregnancy described sharing mental health concerns as “hard” because she did not want to reveal and expose her emotions:

“It’s a personal feeling kind of thing and sometimes it’s hard to share because you don’t like feeling that way or you don’t like feeling you know extra weepy and everything. You don’t like exposing your emotions to people right?” (Participant 12)

#### Perception about prenatal care providers’ role

Some participants perceived maternity care providers as responsible for monitoring only the physical well-being of the mother and the baby. These women considered emotions personal matters and reported that they would not communicate their personal problems. An immigrant multiparous woman indicated that she would feel comfortable discussing any physical concerns with her maternity care provider but that she would communicate mental health concerns only with trusted family members. She commented:

“If I talk … it would be in a professional way, medical things like that. But personal things, I don’t think so. I don’t think so. It’s much better to talk to a person you can trust, loved ones probably.” (Participant 2)

### Structural barriers

Structural barriers included the lack of continuity of care, depersonalized care, lack of feedback, and unfamiliarity with/uncertainty about the availability of support*.*

#### Lack of continuity of care

Several pregnant women reported that seeing one provider at each prenatal care visit would make them feel more comfortable sharing emotional concerns. According to participants, the lack of care continuity hindered their building a rapport with the provider to communicate sensitive issues. A 22-year-old expectant single mother commented:

“If I had the same … [provider] that came in to every single time might inspire me to talk about it [emotional concerns].” (Participant 11)

Trust and sincerity were emerged as important considerations when sharing emotional concerns. In response to the question regarding which qualities would encourage the respondent to share mental health concerns, a multiparous woman stated: “somebody… I can trust... that understands your situation”. A nulliparous woman commented: “just sincerity.”

#### Depersonalized care

Some participants, particularly first-time mothers, reported experiencing an unwelcoming environment. This lack of individual-centered care and medicalized prenatal care discouraged communication around mental health concerns. Feeling like “just another number” was an expression used by a 29-year-old first-time mother with a history of depression. She commented:

“Its their *job* and they know their *job* well. Its just that in your first appointment if they could make it a little bit more personalized as opposed to you’re just another number coming in… but like my initial appointment here and meeting with the nurse wasn’t like ‘hey how are you, how are you doing?’ its just like ‘I’m gonna ask you a whole bunch of questions and you’re gonna tell me as fast as you can. Have you had this, this, this, this and that?’ and I’m just like ‘uh hi. I don’t even know what you guys do here. This is my first pregnancy’. So it would have been a whole lot nicer to be like … ‘I understand that this is your first pregnancy, have you ever had a history of this?’ and trying to talk that into it instead of [snaps fingers 4 times] and out. Goodbye. That would have been better.” (Participant 3)

#### Lack of feedback

Several participants who had previous experiences with screening programs or mental health care services indicated that not receiving feedback discouraged them from engaging or further sharing their mental health concerns. A multiparous woman with history of anxiety disorders stated:

“Well, I think that I should talk to somebody but a lot of time you don’t get a lot of feedback back on it so I don’t know if there’s any growth you actually could sometimes get out of it. I would only learn to do stuff if you get feedback and I’m hoping that these people who I’m hoping to see will give me feedback on it. Like here are some mechanisms for change or coping or something but a counsellor when you go and sit with them, they really just don’t. They really just listen and don’t give anything back so it’s hard because you come away with feeling bad about yourself and no mechanisms of change so...” (Participant 14)

A nulliparous women with history of panic disorders commented:

“Well they’re not really like ... they’re more um it’s just quick in “how are you doing?” and stuff like that “I’m ok” and yeah. It’s not really beneficial, it’s just more to make sure that my head’s in the right place I guess.” (Participant 9)

A need to review screening results and receive feedback or have a follow up appointment after an initial screening also emerged. A participant who had realized she was suffering from anxiety after completing a screening form described that not receiving feedback after screening made her feel lost in the system:

“I think I *was* expressive of what I was going through and emotionally distressed and a nurse probably should have picked up on it. I remember there was a questionnaire that I filled out and I think it would have shown that I had post-partum anxiety… and it never was reviewed so uh I guess it was my chance then to ask for help. I guess I was just waiting for somebody to ask you know, ‘how are you feeling about this? Or would you like support in this area?’…I was waiting for it to be uncovered but no one uncovered it…. It’s really up to women to take up their own healthcare into their hands and seek the help they need because nobody’s really looking after you, I find.” (Participant 7)

#### Unfamiliarity with/uncertainty about the availability of support

Not knowing what support would be available if a diagnosis were ascertained also hindered the disclosure. A pregnant mother who was struggling with uncertainty about the normality of her feelings noted that if she realized her thoughts were not normal, she would need “immediate support.” She commented:

“... if it’s not normal there has to be immediate support rather than “gee I’ll go and sleep on this and oh, my gosh I’m not... I’m not a normal person”. There has to be support... like from what I’ve heard this is not a normal feeling so right now we need you to…” (Participant 12)

Women’s and their partners’ unfamiliarity with available resources and how to receive support for mental health problems also emerged. A 38-year-old multiparous woman who was not aware that she was struggling with anxiety in her previous pregnancy stated that although her partner and friends were aware of her struggles, they did not know how they could identify with and support her:

“I think I saw a lot of friends recognize that I was struggling but didn’t quite know what to do. I know it was difficult for my husband to see me go through it and he didn’t know quite what to do. So in that instance I wish I would have gotten more support.” (Participant 7)

## Discussion

The method of administering screening tools is a component of acceptability for perinatal mental health screening [19]. Acceptability refers to the willingness to complete or administer an instrument or conduct a procedure. There is no uniform psychometrically tested tool to measure acceptability [19]. The research on the acceptability of perinatal mental health screening has mainly focused on examining specific types of screening tools. With few exceptions, evidence on the impact of screening settings (e.g., home, clinic) on women’s comfort [33, 34] or the method of screening is scarce [21, 35]. In Drake et al., postpartum women described online screening as an unintimidating and easy approach to screen for depression that can help in overcoming the challenges of fear and stigma. In our study, while some women favored an online approach as a venue to sustain personal space, others brought up concerns regarding the safety of online screening, time constraints, and a lack of human interaction. Kingston et al. reported paper-based screening as the most comfortable and telephone-based screening as the least comfortable approaches for women [21]. In our study, some participants who preferred “paper-based” screening explained that they did not want to become emotional in the presence of others. Two women explained that this method would give them some time to think and provide “the best answer.” One of these women was suffering from intrusive thoughts of harming her toddler and did not report a previous mental health problem. In our study, women with known mental health issues who had previously interacted with mental health specialists preferred an interactive and in-person screening approach in order to receive feedback. These findings may suggest that women with undiagnosed mental health conditions may choose a less interactive approach and be reluctant to disclose their struggles. Several sources of uncertainty regarding the normality of symptoms, potential consequences of disclosure, and availability of immediate support contributed to the concealment of mental health problems among our participants. Chew-Graham et al. (2009) found that women described making a conscious decision about whether to disclose their feelings to their health care providers [36]. Williams et al. (2016) also reported that while many women would share their emotional issues with their care providers, some would not [20]. Kingston et al. (2015) found that 21 % of women indicated that they could be only somewhat or not at all honest during screening mainly due to fear that their provider would view them as bad mothers [22]. Women in our study reported that a trust relationship and an understanding attitude of the care provider would encourage them to share their concerns. For some women, continuity of care was important in building the trust relationship. A nonjudgmental attitude of the care provider was reported to help women disclose their emotional problems [20].

Uncertainty about potential implications after sharing their thoughts or feelings regarding poor mental health was identified as an important barrier for disclosure by participants. A recent study, similar to ours, found that most participants were unsure about the kind of help that was available and some reported this as a reason for withholding their true feelings [20]. Unclear pathways and protocols may also impact health care providers’ attitudes towards mental health screening [36]. Chew-Graham et al. (2009), in a qualitative study, found that maternity care providers used strategies to hinder disclosure and were reluctant to make a diagnosis of postnatal depression, due to the lack of referral services for further assessment and treatment [36].

Some participants in our study stated that they would be more comfortable to share mental health issues with family members and significant others. This is consistent with previous quantitative research [37], particularly among those in minority groups [38]. In a qualitative study, pregnant women reported that during their prenatal care visits, they expected the assessment of the physical progress of pregnancy and the development of their baby but not their emotional health. This lack of clarity about the role of prenatal care providers made some women feel as if they were “being watched” and feel uncomfortable [39].

In our study, women who initially communicated their mental health concerns voiced a continuous need to receive feedback throughout the process. According to women, receiving feedback was a vital component not only for communicating but also for sustaining their engagement throughout the follow-up process. A lack of feedback was discouraging for our participants as it was viewed as not offering any mechanism for addressing mental health problems.

Mental health literacy is defined as knowledge about mental health symptoms, risk factors, causes, and treatments [32]. Some participants reported that they did not communicate their emotional challenges because they were not able to understand their feelings. Several women in our study also reported that they were not able to differentiate normal and non-normal symptoms despite doubting their mental well-being. Thus, they tried to understand these feelings on their own or hoped that the symptoms would be relieved by taking it “day-by-day”. The findings also highlighted the educational aspect of screening that helped one of the participants realize she was struggling with anxiety. In a recent study, Fonseca (2015) found that the most frequently identified barriers to women’s seeking professional help were related to the level of mental health literacy, followed by practical and structural barriers, such as time and cost constraints, and attitudinal barriers, such as shame and stigma. They reported that over half of the women who screened positive for depression during the perinatal period were unable to recognize the presence of an emotional or psychological problem [14]. Raising the level of public mental health literacy can contribute to early recognition and appropriate intervention-seeking behaviors [32].

Due to the paucity of qualitative evidence on the methods of perinatal mental health screening, one of the strengths of this study is its exploratory nature, which enabled us to understand the underlying reasons for screening preferences. Another strength of the study was conducting two interviews during pregnancy. However, our study has several limitations that require consideration when interpreting and applying the findings. This is a small study in an urban geographical area. Thus, the findings might not be applicable for all settings. The majority of participants in our study were educated, Caucasian, and partnered, and some were recruited from a mental health clinic; these characteristics may also limit the transferability of the findings. To enable clinicians to determine the applicability of the findings to their populations, we provided details of participants’ characteristics in two tables and throughout the paper.

## Conclusions

While the benefits of screening for perinatal mental health outweigh the harms [15], several considerations need to be taken into account for successful screening programs. In the present study, women with known mental health issues preferred an in-person approach while women without a known mental health history who struggled with emotional problems were inclined towards less interactive approaches and reported a reluctance to share their concerns. This is an important finding, as these women are the central target of screening programs but might be missing in the current screening system. Clarity around the outcomes of communicating mental health concerns and the availability of immediate support were perceived by the women as essential for sharing mental health concerns. The overlaps between some themes identified for the reasons behind a preferred screening approach and barriers reported by women to communicate mental health concerns suggest that having options may help women overcome some of the current disclosure barriers and enable them to engage in the process. For example, a lack of trust and not knowing the provider was perceived as a barrier by women. On the other hand, women described that an in-person interactive screening approach would give them an opportunity to know their provider better and make them more inclined to share their concerns. These results may have implications in settings where the continuity of care is not feasible. Based on these findings, the disclosure of mental health struggles requires the availability of multiple screening approaches to address the needs of all women, continuity of care to establish trust, clarity around the outcomes of communicating mental health concerns, availability of immediate support and a system of care with clear pathways to help women move from providing “the best answer” to providing an authentic answer. A culture of maternity care in which asking for help to clarify or understand emotional concerns is perceived safe by women should be promoted to reduce fear of being judged or social interventions. Furthermore, increasing public knowledge about symptoms of perinatal mental health problems and what is considered “normal” can encourage help-seeking behaviors.
